# Obesity and its relationship with lifestyle behaviors among Kuwaiti adolescents revisited after 15 years: a study protocol

**DOI:** 10.3389/fpubh.2026.1808837

**Published:** 2026-05-01

**Authors:** Noura Alosaimi, Abdulaziz Kh. Al-Farhan, Dalal U. Z. Alkazemi, Hazzaa M. Al-Hazzaa

**Affiliations:** 1 Food and Nutrition Administration, Ministry of Health, Kuwait City, Kuwait; 2 Biomedical Science Department, College of Nursing, Public Authority for Applied Education and Training, Kuwait City, Kuwait; 3 Department of Food Science and Nutrition, College of Life Sciences, Kuwait University, Sabah Al-Salem University, Kuwait City, Kuwait; 4 Lifestyle and Health Research Center, Health Sciences Research Center, Princess Nourah Bint Abdulrahman University, Riyadh, Saudi Arabia; 5 School of Sport Sciences, University of Jordan, Amman, Jordan

**Keywords:** accelerometry, adolescent, dietary habit, grip strength, Kuwait, obesity, physical activity, psychological health

## Abstract

**Background:**

Lifestyle behaviors such as physical activity, sedentary time, sleep, and dietary habits play a central role in shaping adolescent health. In Kuwait, the Arab Teens Lifestyle Study (ATLS), conducted in 2009/2010, reported that nearly half of adolescents were overweight or obese, with physical inactivity and unhealthy dietary patterns emerging as key correlates. Over the past 15 years, profound societal changes, including accelerated digitalisation and shifts in food environments, may have further altered these patterns, yet updated nationally representative data are lacking.

**Objectives:**

This study will reassess the prevalence of overweight, obesity, and lifestyle behaviors among Kuwaiti adolescents and directly compare findings with the 2009/2010 ATLS using the same methodological framework. For the first time in Kuwait, psychological wellbeing, grip strength, and objectively measured physical activity and sleep will also be assessed.

**Methods:**

A school-based cross-sectional survey designed as a methodological revisit to the 2009/2010 ATLS will be conducted among a representative sample of 14–19-year-old students from public and private secondary schools across Kuwait using a multistage stratified cluster sampling design (target n ≈ 1,320–1,350). Data collection will include standardized anthropometric measurements, validated questionnaires covering physical activity, sedentary behaviors, sleep, dietary habits, and psychological wellbeing, grip strength dynamometry, and wrist-worn accelerometery where funding permits. All inferential analyses will be conducted using survey-adjusted procedures accounting for the complex sampling design.

**Expected outcomes:**

This study will generate nationally representative, contemporary evidence on adolescent health behaviors in Kuwait, enable direct temporal comparisons with 2009/2010 baseline findings, and support the development of targeted public health strategies aimed at reducing obesity and improving the wellbeing of Kuwaiti adolescents.

## 1 Introduction

Non-communicable diseases, including cardiovascular disease, diabetes, and several cancers, remain the dominant health challenge of the 21st century and their behavioral determinants are well established (1). Modifiable lifestyle patterns such as inadequate physical activity, prolonged sedentary time, poor dietary quality, and insufficient sleep contribute to obesity, metabolic dysregulation, and psychological distress (1, 2). Longitudinal evidence consistently demonstrates that individuals who engage in regular physical activity, follow balanced dietary patterns rich in fruits and vegetables, and maintain adequate sleep exhibit lower chronic disease risk and better overall wellbeing (3). While these relationships are well recognized, less is known about how such behaviors shift across time within rapidly changing societies.

Adolescence marks a formative period during which lifestyle behaviors consolidate and track into adulthood (4). In Kuwait, previous research has documented high rates of adolescent obesity and physical inactivity, identifying this age group as a key priority for early intervention (5, 6). The WHO Global Action Plan on Physical Activity 2018–2030 emphasizes the need to support young people and reverse rising inactivity trends, aiming for a global 15% reduction in physical inactivity by 2030 (7). Evidence shows that adolescents who develop healthier movement, sleep, and dietary behaviors experience benefits that extend into adulthood (8). Understanding these behaviors in Kuwait is therefore essential to designing targeted, youth-focused health promotion strategies.

The most comprehensive population-level evidence on Kuwaiti adolescent lifestyle behaviors to date derives from the Kuwait component of the Arab Teens Lifestyle Study (ATLS), a multicentre school-based cross-sectional study conducted in 2009/2010 among 906 randomly selected adolescents aged 14–19 years, drawn from all six governorates using a multistage stratified cluster sampling design (29). Its findings painted a concerning picture of adolescent health in Kuwait. The prevalence of overweight and obesity was 50.5% in boys and 46.5% in girls (29). Nearly half of boys (44.6%) and three-quarters of girls (76.0%) did not meet recommended daily physical activity levels, while screen time exceeded 2 h per day in 96.3% of boys and 96.7% of girls (29). Sleep insufficiency was widespread, with approximately 76% of boys and 74% of girls sleeping 6 h or fewer per night. Dietary habits were equally concerning: the majority of adolescents reported skipping breakfast and infrequent consumption of fruits, vegetables, and dairy, alongside frequent consumption of fast food and sugar-sweetened beverages (29). Physical inactivity and poor dietary habits were identified as the strongest correlates of overweight and obesity in this population (29).

Subsequent studies have confirmed that these patterns have persisted. The Study of Health and Activity Among Adolescents in Kuwait (SHAAK), conducted in 2012/2013 using objective accelerometery, found that only 3.4% of adolescents met physical activity guidelines when objectively measured, and approximately 60% of boys and 48% of girls were overweight or obese (11). More recently, Al-Haifi et al. reported an obesity and overweight prevalence approaching 50%, with males disproportionately affected (5). These figures underscore the scale and persistence of the problem and highlight the urgent need for updated nationally representative surveillance data.

Kuwait presents a particularly dynamic context. Over the past 15 years, adolescents' daily lives have been transformed by accelerated digitalisation, shifts in food environments, increased reliance on delivery-based meals, and prolonged engagement with screen-based platforms. Although obesity remains highly prevalent, with estimates frequently approaching 50% (5), current evidence relies largely on small, convenience-based or governorate-specific samples, limiting the ability to identify national trends or emerging behavioral patterns (5, 6, 12). Existing data therefore do not capture how contemporary lifestyle behaviors compare with those observed in the original ATLS, prior to these major societal changes.

Additionally, key determinants that are now recognized as central to adolescent health, such as psychological wellbeing, sleep quality, and muscular strength, were not included in the original ATLS. Evidence linking sleep duration, diet, and sedentary behavior to mental health symptoms in adolescents (13), and emerging research identifying grip strength as a marker of metabolic health, underscore the need for broader, multidimensional assessment. Likewise, as the SHAAK study demonstrated, objective measurement of physical activity and sleep using accelerometery reveals markedly higher inactivity rates than self-report alone, yet this approach has not been implemented in a nationally representative adolescent sample in Kuwait since that study was conducted over a decade ago.

The present study therefore revisits adolescent obesity and its relationship with lifestyle behaviors 15 years after the baseline ATLS, using an identical sampling framework and methodological approach to enable valid temporal comparisons. By additionally incorporating psychological wellbeing, grip strength, and objective assessments of movement behaviors through accelerometery, the study captures both continuity and change across a period marked by profound societal transformation. It addresses a critical surveillance gap by providing updated, nationally representative evidence on how Kuwaiti adolescents' health behaviors have evolved, evidence that is essential for developing precise, contemporary health promotion strategies.

**Summary of Aims:** This study aims to: (1) reassess the prevalence of overweight, obesity, and key lifestyle behaviors among Kuwaiti secondary school adolescents aged 14–19 years, and compare current findings directly with those from the 2009/2010 Kuwait ATLS using the same sampling framework and methodological approach; (2) examine, for the first time in a nationally representative Kuwaiti adolescent sample, the associations between obesity and psychological wellbeing, grip strength, and objectively measured physical activity and sleep; and (3) explore the influence of sociodemographic factors on these health indicators. The specific primary and secondary objectives and research questions guiding the study are presented below.

The primary aim of this research project is to collect comprehensive and contemporary data on obesity and key lifestyle behaviors, including physical activity, sedentary time, sleep, and dietary habits, alongside measures of grip strength and psychological wellbeing among Kuwaiti adolescents attending secondary schools. A further aim is to directly compare current levels of overweight/obesity, physical inactivity, sedentary behaviors, dietary patterns, and sleep duration with data collected 15 years ago as part of the Kuwait component of the ATLS, using the same sampling design and methodological framework (9, 29) to ensure methodological continuity and comparability over time.

For the first time in Kuwait, this study will additionally incorporate validated assessments of psychological wellbeing and grip strength, together with objective measurements of physical activity, sedentary time, and sleep using accelerometers (subject to funding availability). Integrating these novel components will expand the scope of adolescent health surveillance in Kuwait and provide deeper insights into the broader behavioral and physiological determinants of adolescent health.

### 1.1 The primary objectives of the ATLS revisited research project are to:

1. Examine the current prevalence of overweight, obesity, and abdominal obesity among Kuwaiti adolescents, and compare these estimates with data collected approximately 15 years ago.
2. Assess current lifestyle behaviors, including physical inactivity, sedentary behavior, sleep duration, and healthy and unhealthy dietary habits, and compare these patterns with those documented in the earlier ATLS.
3. Evaluate secular changes in overweight/obesity, physical activity, sedentary behavior, sleep duration, and dietary patterns among adolescents in Kuwait, and assess current indicators of physical and mental health.

### 1.2 The secondary objectives of the ATLS revisited research project are to:

4 Examine the associations of both physical and mental health indicators and key lifestyle behaviors (physical activity, sedentary time, sleep duration, and dietary habits).
5 Explore the influence of sociodemographic factors on obesity, lifestyle behaviors, and overall health, including physical and psychological wellbeing.

### 1.3 Research questions (organized by thematic domains)

#### A. Prevalence and trends

6 What is the current prevalence of overweight, obesity, and abdominal obesity among Kuwaiti adolescents, and how have these estimates changed compared with the ATLS conducted 15 years ago?
7 What are the current prevalence levels of physical inactivity, sedentary behavior, and insufficient sleep, and how do they compare with patterns reported in 2010–2011?
8 How have healthy dietary behaviors (e.g., breakfast intake, fruit, vegetable, and dairy consumption) and unhealthy dietary habits (e.g., fast food, fried foods, sugar-sweetened beverages, and energy drinks) evolved since the earlier ATLS?

#### B. Objective measures of movement behaviors

9 What is the prevalence of physical inactivity, sedentary behavior, and insufficient sleep among Kuwaiti adolescents when assessed objectively using ActiGraph accelerometers?

#### C. Behavioral and psychological determinants of health

10 Which lifestyle behaviors and dietary habits are most strongly associated with obesity, abdominal obesity, and psychological wellbeing among Kuwaiti adolescents?
11 How are breakfast intake, food preferences, and other eating behaviors related to overweight and obesity status?
12 How do sociodemographic and socioeconomic factors influence lifestyle behaviors, obesity risk, and psychological wellbeing in adolescents?

#### D. Muscular strength and physical health indicators

13 What are the associations between grip strength and physical activity, sedentary behaviors, and adiposity in this population?
14 Which lifestyle behaviors, dietary habits, and measures of muscular strength are the strongest predictors of obesity and abdominal obesity among Kuwaiti adolescents?

#### E. Gender-specific patterns

15 Are there gender differences in obesity, lifestyle behaviors, eating habits, grip strength, or psychological wellbeing, and if so, what is the magnitude of these differences?

The study will collect a comprehensive set of anthropometric measurements across the sample, including weight, height, sitting height, waist circumference, BMI, waist-to-height ratio, and grip strength. Demographic and socioeconomic information, such as age, sex, family characteristics, type of school (public vs. private), and mode of transportation to and from school, will also be recorded. Key lifestyle factors will be assessed through validated instruments and will include levels of physical activity, sleep duration, screen time, and perceived barriers and facilitators to engaging in physical activity.

Selected outcome measures will be used to generate a detailed profile of adolescent health behaviors and status. These include:

- The prevalence of overweight, obesity, and abdominal obesity among Kuwaiti adolescents, disaggregated by age and sex, with additional comparisons across early, mid, and late adolescence (using chronological age or maturity status).
- The proportion of adolescents meeting vs. not meeting physical activity guidelines, stratified by gender.
- The percentage of adolescents exceeding recommended thresholds for sedentary behavior.
- The prevalence of sufficient vs. insufficient sleep duration on weekdays and weekends, stratified by sex.
- The proportion of adolescents who regularly skip breakfast, with weekday–weekend comparisons.
- The percentage of adolescents reporting healthy dietary habits, including regular intake of fruits, vegetables, and dairy products.
- The proportion of adolescents reporting unhealthy dietary behaviors, such as frequent consumption of fast food, fried foods, and sweetened or ultra-processed snacks.

These indicators will enable a nuanced understanding of adolescent lifestyle patterns and their variation across demographic groups.

To extend and modernize the original ATLS framework, the present study will incorporate several new measures that provide a broader and more comprehensive understanding of adolescent health:

- Objective assessments of physical activity, sedentary time, and sleep using wrist-worn accelerometers (subject to funding availability).
- Measures of psychological wellbeing, capturing emotional, behavioral, and stress-related domains relevant to adolescent health.
- Grip strength assessment as an additional marker of muscular fitness and its relationship with cardiometabolic risk and functional health.

Adolescent obesity remains one of Kuwait's most pressing public health challenges, with nearly half of young people affected—particularly males—at levels that exceed global averages (5). Establishing the current prevalence of overweight, obesity, and abdominal obesity, and comparing these estimates with findings from the ATLS conducted 15 years ago, will enable the identification of persistent gaps as well as emerging risk patterns. This comparison is essential: childhood and adolescent obesity strongly predict metabolic complications in adulthood, increasing the long-term burden on healthcare systems (14). By generating an updated, nationally representative profile of adolescent health, the study provides crucial evidence to guide early intervention strategies capable of altering obesity trajectories and preventing long-term complications.

Another key justification for this research is the absence of robust national data on adolescent dietary habits and nutritional status in Kuwait. The Kuwait Nutrition Surveillance System (KNSS), despite being a primary source of population-level nutrition data, offers minimal detail on adolescents' dietary patterns, obesity determinants, or lifestyle behaviors. This major surveillance gap leaves policymakers with limited evidence on the nutritional challenges facing youth. Addressing this gap is essential for developing adolescent-specific strategies within Kuwait's broader nutrition policy framework.

Beyond reassessing prevalence, this study provides a broader and more integrated understanding of adolescent health than previously available in Kuwait. The inclusion of psychological wellbeing, grip strength, and objective movement data captured through accelerometers introduces health dimensions that were not assessed in the original ATLS. Only a small number of local studies have used ActiGraph devices, and the existing evidence is both dated and limited in scope. The SHAAK study (11), for example, relied on a relatively small sample drawn from only three governorates and was conducted more than a decade ago using earlier-generation accelerometers. Another accelerometer-based study (15) focused mainly on adults with type 1 diabetes, limiting the generalisability of its findings to the wider population.

Incorporating objective measures and novel indicators in the present study will produce high-quality behavioral data that better capture the complexity of adolescents' daily activity patterns. Additionally, examining the relationship between nutrition literacy and obesity is especially relevant in Kuwait, where nutritional knowledge does not always translate into healthier behaviors (16). Understanding how awareness, behavior, and health outcomes interact within this cultural context will provide valuable insights into barriers to effective behavior change.

Overall, the significance of this study lies in its ability to deliver a comprehensive, multidimensional, and contemporary assessment of adolescent health in Kuwait—directly addressing national data gaps left by the KNSS and extending beyond the scope of the original ATLS. The findings will support the development of targeted, evidence-based interventions and policies that reflect the realities of Kuwaiti adolescents and contribute to reducing the long-term burden of obesity and related chronic diseases.

## 2 Methods and analysis

### 2.1 Study design

This study is a school-based cross-sectional survey designed as a methodological revisit to the Kuwait component of the 2009/2010 Arab Teens Lifestyle Study (ATLS). A new cohort of Kuwaiti adolescents is recruited from the same population frame using an identical sampling strategy, eligibility criteria, instrumentation, and measurement protocol applied 15 years earlier. This deliberate methodological continuity—rather than *post-hoc* harmonization—is the primary mechanism enabling valid population-level temporal comparisons. Findings will be interpreted as secular changes in prevalence and behavior at the population level within the same national sampling frame.

### 2.2 Ethical approvals

Ethical approval will be obtained from the Ministry of Health in Kuwait. Approval to conduct research in schools will be sought from the Ministry of Education and from principals of all participating schools. Because participants are minors, written parental consent will be obtained for all students under 18 years of age, and written assent will be obtained from the adolescents themselves. Students aged 18 years and older will provide their own written informed consent. All participants will be informed that participation is voluntary and that they may withdraw at any time without penalty. Procedures will follow the Declaration of Helsinki. Data will be de-identified, coded, and stored securely with restricted access. No physical, emotional, or social harm is anticipated.

### 2.3 Participant eligibility criteria

Eligible participants are Kuwaiti adolescents enrolled in grades 10–12 (ages 14–19 years).

#### 2.3.1 Exclusion criteria include:

- medical conditions affecting normal eating patterns (e.g., food allergies requiring avoidance, diagnosed eating disorders),
- medical conditions that prevent safe participation in physical activity,
- physical disabilities that prevent completion of anthropometric measurements.

### 2.4 Sample size and sampling technique

To ensure comparability with the 2009/10 Kuwait ATLS (29), the same multistage stratified random sampling strategy will be used. For reference, the 2009/2010 Kuwait ATLS recruited a total of 906 adolescents (463 boys and 443 girls) aged 14–19 years from secondary schools across all six governorates using the same sampling framework (9). The present study targets approximately 1,320–1,350 participants, representing a substantially larger and more powered sample to enable precise sex-specific estimates and subgroup analyses not possible with the original dataset.

Schools will be stratified by sex (boys/girls), sector (public/private), and governorate. From each governorate, one boys' school and one girls' school will be randomly selected (12 public schools total). Within each school, one randomly selected class from grades 10, 11, and 12 will be included, resulting in 36 public school classes. An additional six classes will be randomly selected from private schools, proportional to enrolment, yielding 42 classes in total.

School sector allocation will be proportional to national enrolment figures obtained from the Kuwait Ministry of Education for the study year. Based on current enrolment data, approximately 75–80% of Kuwaiti secondary school students attend public schools and 20–25% attend private schools; the sampling frame will reflect this distribution, with private school classes selected proportionally to their share of total enrolment within each governorate. This ensures the achieved sample is representative of the national secondary school population by sector.

Class sizes typically range from 30–35 students. With an anticipated participation rate of 70–75%, approximately 25 students per class are expected to take part, yielding approximately 1,200 participants.

#### 2.4.1 Sample size calculation

Using Cochran's formula for proportions with *p* = 0.50, *Z* = 1.96, and *d* = 0.04, the required sample size is approximately 600. To account for cluster sampling and ensure precise sex-specific estimates, a design effect of 1.5 (ICC≈0.02; cluster size≈25) yields an effective sample of approximately 800 (≈400 per sex). This requires approximately 1,200 completed responses (800 × 1.5). Allowing 10% additional recruitment to offset missing data, the target sample is approximately 1,320–1,350 adolescents.

An *a priori* G^*^Power analysis indicated that 129 participants would be sufficient to detect a small effect size (*f*^2^ = 0.02) with four predictors at α = 0.05 and 95% power. The planned sample (>1,300) provides ample power to detect even small associations.

### 2.5 Measurement procedures

Table 1 summarizes the variables and assessment methods.

**Table 1:** Study variables and methods of assessment.

Assessed variable	Assessment method
Anthropometric measurements (weight, height, sitting height, BMI, waist circumference, WHtR)	Direct measurement using standardized procedures
Demographics and SES (age, parental education, maternal education, income, GPA, school type)	Self–reported
Physical activity	Self–reported (ATLS); objective accelerometery (if funded)
Sedentary behaviors	Self–reported (ATLS/ASBQ); accelerometery (if funded)
Sleep duration	Self–reported (ATLS); accelerometery (if funded)
Dietary habits	Self–reported (ATLS FFQ, validated for adolescents)
Psychological wellbeing	Self–reported (BPWBS, validated for adolescents)
Grip strength	Direct measurement using calibrated dynamometer
Biological maturation	Maturity offset method (anthropometric-based equation)

#### 2.5.1 Anthropometric measurements

Anthropometric assessments will include weight, height, sitting height, BMI, waist circumference (WC), and waist-to-height ratio (WHtR). Measurements will be taken in the morning by trained researchers following standardized procedures, ensuring privacy.

- Weight: measured to the nearest 0.1 kg using a calibrated scale, with light clothing and no shoes.
- Height: measured to the nearest 0.1 cm using a stadiometer, without footwear.
- Sitting height: measured to the nearest 0.1 cm using a stadiometer with the participant seated on a flat, stable surface, hips and knees flexed at 90°, back straight, and head positioned in the Frankfurt plane.
- BMI: calculated as kg/m^2^; classified using IOTF age- and sex-specific cut-offs for 14–17-year-olds (37) and WHO adult cut-offs for those ≥18 years.
- Waist circumference: measured to the nearest 0.1 cm, 1 cm below the navel, at end-expiration.
- WHtR: calculated as WC (cm) ÷ height (cm); WHtR ≥ 0.50 will define abdominal obesity in both sexes (17, 18).

#### 2.5.2 Demographics and socioeconomic status

Demographic and socioeconomic data will include age, parental and maternal education, parental employment, household income (categorized as: < 1,000; 1,000–1,500; 1,501–2,000; 2,001–2,500; 2,501–3,000; 3,001–3,500; 3,501–4,000; >4,000 KWD), and type of school. These factors will be examined given their known associations with adolescent lifestyle behaviors and obesity. Information on the number of children under 18 living at home will also be collected, as family structure may influence lifestyle habits (19).

#### 2.5.3 Biological maturation

Biological maturation will be estimated using the maturity offset method (10), which predicts years from peak height velocity (PHV) based on standing height, sitting height, weight, and age. All of these measures are collected as part of the standard anthropometric protocol, requiring no additional participant burden.

Although the Pubertal Development Scale (PDS) (39) has been used in large-scale international adolescent surveys, self-reported questions about pubertal development are culturally sensitive in conservative Arab societies such as Kuwait, where discussion of bodily changes is considered private. Including such items in a school-administered questionnaire risks reducing adolescent and parental participation and introducing non-response bias, both of which would compromise the national representativeness that is a primary strength of this protocol. The maturity offset method, being derived entirely from objective anthropometric measurements, avoids these concerns entirely.

Maturity offset scores will be categorized into broad groupings of early, mid, and late maturity rather than treated as a continuous variable, minimizing the sensitivity to prediction error known to affect the method, particularly at the extremes of the maturity distribution. Pubertal status will be treated as a covariate in all regression models rather than a primary outcome. The potential for prediction error in applying these equations to an Arab adolescent population is acknowledged as a study limitation.

#### 2.5.4 Physical activity

Physical activity will be assessed using the Arab Teens Lifestyle Study (ATLS) questionnaire, which has demonstrated strong validity and reliability for adolescents and young adults aged 14–25 years (9, 20, 31). The tool captures frequency, duration, and intensity of light, moderate, and vigorous activities across various domains, including commuting, household chores, structured sports, and leisure-time physical activity.

From participants' responses, the following indicators will be computed:

- Total weekly physical activity (minutes per week),
- Time spent in moderate-to-vigorous physical activity (MVPA),
- Total energy expenditure expressed as MET-minutes per week.

MET values will be assigned using standardized compendia for youth and adults (34, 36). To estimate the proportion of adolescents meeting physical activity guidelines, two thresholds will be applied:

- ≥60 min/day of moderate-intensity activity (4 METs), equivalent to 1,680 MET-minutes/week.
- ≥60 min/day of MVPA (6 METs), equivalent to 2,520 MET-minutes/week.

In addition, those reporting ≥420 min/week of moderate-intensity activity will be classified as meeting recommendations (38). These thresholds will be used to identify the prevalence of sufficiently active vs. insufficiently active adolescents. Participants will also report their primary mode of transport to and from school (e.g., walking, car, bus), to capture active commuting behavior.

#### 2.5.5 Sedentary behaviors

Sedentary time will be assessed through the ATLS questionnaire, which includes self-reported screen-based activities such as TV viewing, video gaming, computer use for leisure, and internet browsing. Separate estimates will be collected for weekdays and weekends.

Sedentary behavior will be classified using a < 2 h/day cutoff for recreational screen time, based on American Academy of Pediatrics guidelines, and an additional threshold of ≥3 h/day to identify high screen users (40).

To obtain a more comprehensive estimate of sitting time, the Arabic Sedentary Behavior Questionnaire (ASBQ) will also be administered. The ASBQ captures sitting time across 13 activities, differentiating between weekday and weekend patterns. It has demonstrated strong reliability (κ = 0.95) and content validity in Arab populations (35).

#### 2.5.6 Sleep duration

Sleep duration will be assessed using items from the ATLS questionnaire, with participants reporting average hours of sleep on school days and weekends. Adolescents aged 14–17 years sleeping < 8 h/night, and those aged 18–19 years sleeping < 7 h/night, will be classified as short sleepers, in accordance with the National Sleep Foundation's age-specific recommendations (21).

#### 2.5.7 Dietary habits and intake

Dietary behaviors will be assessed using the ATLS food-frequency section, which includes 10 items on weekly consumption of common foods and beverages. Participants will report how many days per week they typically consume breakfast, fresh and cooked vegetables, fruits, milk and dairy products, sugar-sweetened beverages, fast foods, cakes/donuts, sweets/chocolates, fried snacks (e.g., French fries or potato chips), and energy drinks. Response options range from “never” (0 days/week) to “daily” (7 days/week). This tool has demonstrated acceptable validity for adolescents in the region (22) and is retained primarily to ensure direct comparability with the 2010 ATLS dataset.

**Dichotomisation strategy:** Dietary variables will be dichotomised following the original ATLS methodology to maintain comparability with 2010 findings. Healthy dietary behaviors, breakfast, fruits, vegetables, and dairy products—will be dichotomised as daily vs. non-daily intake, reflecting public health recommendations for daily consumption of these food groups. Unhealthy dietary behaviors, fast foods, sugar-sweetened beverages, fried snacks, sweets/chocolates, and cakes/donuts, will be categorized as less than 4 days/week vs. 4 or more days/week, representing a clinically meaningful threshold for frequent consumption of energy-dense, nutrient-poor foods. While dichotomisation reduces statistical power and loses distributional information, this approach is justified by the overriding need for methodological continuity with the 2010 ATLS. For all novel analyses not requiring historical comparison, dietary variables will additionally be examined in their continuous or ordinal form to maximize analytical sensitivity.

To capture family-level determinants of eating and activity patterns, adolescents will also complete the Family Nutrition and Physical Activity (FNPA) questionnaire (23), a validated 20-item tool assessing family routines related to diet, physical activity, screen time, and sleep. Higher scores indicate more supportive family environments for healthy behaviors.

**Automated multiple pass method (AMPM) subsample:** Although the ATLS dietary questions allow comparison with historical data, they do not capture portion size, total energy intake, or nutrient quality. To address this limitation, the Automated Multiple Pass Method (AMPM), a structured, interviewer-administered 24-h dietary recall designed to minimize under-reporting and improve accuracy, will be administered to a purposively selected subsample of approximately 200 participants (approximately 100 boys and 100 girls) (24). Subsample selection will be stratified by sex, governorate, and school sector (public/private) to ensure adequate demographic coverage and reflect the distribution of the full study sample. This subsample size is consistent with published school-based studies employing the AMPM and is sufficient to generate reliable sex-specific estimates of mean energy and macronutrient intake.

To assess subsample representativeness, baseline sociodemographic and anthropometric characteristics of AMPM participants will be compared with the full study sample. Any meaningful differences will be documented and accounted for in the interpretation of subsample findings. Results derived from the AMPM subsample will be clearly distinguished from full-sample findings and will not be generalized to the national population without appropriate qualification.

#### 2.5.8 Psychological wellbeing

Psychological wellbeing will be assessed using the Brief Psychological WellBeing Scale (BPWBS), a validated short form of the original psychological wellbeing instrument (25). The BPWBS evaluates positive dimensions of mental health, including interpersonal relationships, sense of mastery, and overall emotional functioning. The scale includes 10 items rated on a 5-point Likert scale (1 = strongly disagree to 5 = strongly agree), yielding a total score ranging from 10 to 50. Scores will be categorized as: Very low (10–30), Low (31–37), Average (38–40), High (41–44), and Very high (45–50). The BPWBS has been translated into Arabic using standard forward–backward translation procedures, and its psychometric properties in Arabic-speaking populations are well established, demonstrating high internal consistency (Cronbach's α = 0.91), strong construct validity (CFA = 0.947), and acceptable content validity (25).

#### 2.5.9 Grip strength

Handgrip strength will be assessed as an indicator of upper-body muscular fitness using a digital hand dynamometer (Takei, Japan) (26). Muscular strength is increasingly recognized as a key marker of adolescent health, with evidence linking lower grip strength to insulin resistance, impaired glucose metabolism, and adverse cardiometabolic profiles (27). Studies in adolescents have also shown that both absolute grip strength and measures adjusted for body size are associated with elevated cardiometabolic risk, particularly among males.

The following variables will be recorded: absolute grip strength (kg), grip strength relative to body weight (kg/kg), and grip strength adjusted for BMI or height^2^ to account for body-size differences (28). Participants will stand upright, hold the dynamometer in their dominant hand, and squeeze with maximal effort keeping the arm straight. Each participant will complete three trials with a brief rest between attempts; the highest value will be used in the analysis. Grip strength will be examined as both an independent indicator of muscular fitness and as a potential predictor or correlate of adiposity, physical activity, and psychological wellbeing.

#### 2.5.10 Objective measurement of physical activity, sedentary behavior, and sleep

In addition to self-reported data, physical activity, sedentary behavior, and sleep will be objectively measured using a wrist-worn accelerometer (ActiGraph wGT3X-BT; ActiGraph LLC, Pensacola, FL, USA), a research-grade device widely used in adolescent surveillance studies (41).

**Device initialization and sampling frequency:** Devices will be initialized to collect continuous raw triaxial acceleration data at 100 Hz, consistent with current best practice recommendations for raw data accelerometery in adolescent research and with the requirements of the GGIR processing pipeline.

**Raw data processing:** Raw acceleration data will be downloaded in .gt3x format and processed using the validated open-source R package GGIR (version 2.9+). The processing pipeline will include: (1) auto-calibration of raw signals using the local gravity method; (2) non-wear detection based on standard deviation and value range of the raw triaxial signal; (3) signal cleaning and artifact removal; (4) calculation of the Euclidean Norm Minus One (ENMO), expressed in milligravity units (mg), averaged over 5-second epochs; and (5) computation of the Monitor-Independent Movement Summary (MIMS) as an additional harmonization metric. This raw-data approach avoids proprietary count-based cut-point dependency and supports methodological transparency.

**Valid wear day criteria:** A valid day will be defined as ≥10 h of wear time after removal of non-wear periods. Participants providing ≥4 valid days, including at least 1 weekend day, will be retained for physical activity and sedentary behavior analyses. For sleep analyses, a minimum of ≥3 valid nights will be required. These thresholds are consistent with established protocols in adolescent accelerometery research.

**Sleep detection algorithm:** Sleep parameters will be derived using GGIR's validated HDCZA (Heuristic algorithm looking at Distribution of Change in Z-Angle) sleep detection algorithm, which has demonstrated acceptable agreement with polysomnography in adolescent samples. Sleep outcomes will include total sleep duration, sleep efficiency, and sleep onset and wake times, reported separately for school days and weekends.

**Wear protocol:** Participants will wear the accelerometer on the non-dominant wrist continuously for seven consecutive days, including school days and weekends, removing the device only for contact sports or prolonged water immersion. A wear diary will be provided to log any periods of device removal.

**Non-compliance strategy:** Trained fieldwork staff will provide standardized verbal and written instructions on correct wear protocol prior to deployment. At device collection, compliance will be assessed by inspecting wear patterns both visually and algorithmically within GGIR. Participants with fewer than 4 valid days will be excluded from accelerometery-based analyses but retained for questionnaire-based analyses. Sensitivity analyses will compare sociodemographic and anthropometric characteristics of participants with and without valid accelerometery data to assess potential non-compliance bias.

**Derived outcomes:** Accelerometery-derived outcomes will include average daily ENMO (mg); time spent in sedentary behavior, light, moderate, and vigorous physical activity; and sleep duration, efficiency, and timing parameters derived from the HDCZA algorithm.

### 2.6 Fieldwork team and responsibilities

To initiate recruitment, the research team will contact secondary schools across the six governorates through an official letter issued via the Ministry of Education. The letter will introduce the study, outline its objectives, describe the expected involvement of school staff and students, and request permission to conduct the research. After this initial communication, a member of the research team will schedule a brief in-person or virtual meetings with each school's principal or designated coordinator to explain study procedures, clarify logistical requirements, and confirm participation.

For schools agreeing to take part, the research team will coordinate directly with administrators to schedule suitable dates for on-site visits. Prior to each visit, schools will receive an information pack containing study details, timelines, and copies of adolescent assent forms and parental consent forms.

Two fieldwork teams will be established to support efficient and culturally appropriate data collection. Each team will be led by a co-investigator and supported by three trained researchers, including at least one nurse. One team will collect data in boys' schools and the other in girls' schools, ensuring adherence to cultural guidelines in Kuwaiti educational settings and facilitating parallel data collection across governorates.

During each school visit, the field team will:

- Coordinate with school leadership to organize selected classes and obtain attendance lists.
- Introduce the study to students, verify assent and parental consent, and address questions.
- Administer questionnaires and provide standardized instructions.
- Deploy wrist-worn accelerometers, ensuring correct placement and recording device ID and serial numbers.
- Conduct anthropometric measurements (height, sitting height, weight, waist circumference), grip strength testing, and other assessments following standardized protocols.
- Provide written and verbal instructions on accelerometer use, wear protocol, and return procedures.
- Collect accelerometers after the 7-day wear period, verify compliance, and record any deviations or device issues.
- Manage devices post-collection (downloading data, recharging, re-initializing, and documenting device status) to prepare for the next deployment cycle.

With two parallel teams, all six governorates can be covered within the planned 12–14 weeks with high consistency and data quality.

Kuwait's extreme climate with summer temperatures regularly exceeding 45 °C between June and September significantly constrains outdoor physical activity and alters sleep patterns and dietary behaviors during this period. Data collection will therefore be conducted exclusively during the autumn and winter school terms (October to February), when temperatures are moderate and adolescents' daily routines are most representative of their typical year-round behaviors. Specifically, assessments will not occur immediately after long holidays, during national examination periods, during Ramadan, or in the first 10 days following the return to school after Ramadan, due to substantial shifts in sleep, eating behaviors, and activity patterns during these periods.

### 2.7 Data management plan

All questionnaire responses will be collected electronically through the JISC Online Survey platform, which provides secure data capture, encrypted transmission, and built-in quality control features. Data will be exported directly into standard formats (Excel, CSV, SPSS/Stata) for analysis. Each participant will be assigned a unique identification code to maintain confidentiality and to enable linkage across datasets (questionnaires, anthropometric measurements, grip strength, and accelerometer data).

Data cleaning procedures will be conducted prior to analysis. Questionnaire entries will be checked for completeness, logical consistency, and out-of-range values. Anthropometric and accelerometer data will undergo separate validation checks to identify measurement errors, extreme values, or device malfunctions. Cases with missing key variables will be flagged, and appropriate handling strategies, such as sensitivity analyses or multiple imputation, will be applied where justified. A detailed data management log will be maintained to document all steps and ensure auditability and reproducibility.

All accelerometer data will be downloaded in .gt3x format and processed using ActiLife software (ActiGraph LLC, Pensacola, FL, USA). No personal identifiers will be stored within ActiLife; all device files will be labeled using study codes only. ActiGraph systems conform to international data protection and medical device standards, including GDPR, the EU–US Data Privacy Framework, and ISO 13485:2016. All study files will be retained only for the duration necessary to meet research, reporting, and ethical requirements. After the retention period, data will be securely deleted following institutional and Ministry of Health guidelines.

### 2.8 Pilot study

A pilot study will be conducted with approximately 25 male and female students recruited from a mix of public and private secondary schools in Kuwait. The purpose of the pilot is to test and refine all data collection procedures, assess the clarity and feasibility of the questionnaires, and estimate the time required to complete the full assessment protocol. The pilot will include administration of the full questionnaire set, anthropometric measurements, grip strength testing, and collection of brief feedback from students on clarity, burden, and acceptability of the tools. The full pilot assessment is expected to take approximately 20–25 min per student. Necessary adjustments to the protocol will be made before launching the main study.

### 2.9 Data and statistical analysis

All data will be cleaned and analyzed using Stata version 18 (StataCorp LLC, College Station, TX). All inferential analyses will be conducted using survey-adjusted procedures (Stata svy commands) to account for the multistage stratified cluster sampling design, incorporate post-stratification weights, and produce design-consistent standard errors and confidence intervals.

**Temporal comparisons with the 2010 ATLS:** To ensure valid temporal comparisons with the 2010 ATLS, the same variable definitions, cut-points, and classification criteria will be applied consistently across both datasets (e.g., IOTF BMI cut-offs, physical activity thresholds, dietary dichotomisation rules). Age- and sex-standardized prevalence estimates will be computed where cohort composition differs. Where any demographic shifts are identified between the two time points, regression-based adjustment will be applied. Effect sizes and 95% confidence intervals will be reported for all between-cohort comparisons.

**Survey weighting and cluster adjustment:** Post-stratification weights will be computed based on official Ministry of Education enrolment statistics, ensuring estimates are representative of the national population of Kuwaiti secondary school adolescents by sex, governorate, and school sector. The design effect and intraclass correlation coefficient (ICC) will be reported to document the extent of clustering in the data.

**Primary and secondary outcomes:** Primary outcomes are: (1) prevalence of overweight and obesity classified by IOTF and WHO BMI cut-offs; (2) prevalence of abdominal obesity defined by WHtR ≥ 0.50; (3) proportion of adolescents meeting physical activity guidelines (≥60 min/day MVPA); and (4) proportion exceeding recreational screen time thresholds (≥2 h/day). Secondary outcomes include sleep duration classification, dietary behavior indicators (healthy and unhealthy), psychological well-being scores, and grip strength measures.

**Analytical hierarchy:** Analyses will proceed in three pre-specified stages. Stage 1: descriptive statistics and weighted prevalence estimates, computed separately by sex and compared with 2010 ATLS figures. Continuous variables will be assessed for normality and summarized as means ± SD or medians (IQR) as appropriate. Stage 2: unadjusted bivariate analyses using *t*-tests, ANOVA, chi-square tests, or non-parametric equivalents as appropriate, all survey-adjusted. Stage 3: multivariable regression models, with logistic regression for binary outcomes (overweight/obesity, abdominal obesity, insufficient sleep, high sedentary time) and linear regression for continuous outcomes (grip strength, ENMO-derived activity metrics, psychological wellbeing scores, anthropometric measures). All multivariable models will be adjusted for age, sex, school sector (public/private), household income, parental education, and pubertal status (early/mid/late). Interaction terms (e.g., sex × physical activity) will be explored where theoretically justified.

**Multiple comparisons:** For confirmatory analyses involving primary outcomes, Bonferroni correction will be applied within families of related comparisons. For exploratory secondary analyses, the Benjamini-Hochberg false discovery rate (FDR) procedure will be used, and all such findings will be explicitly labeled as exploratory. Effect sizes (Cohen's d, odds ratios with 95% CI, or partial eta-squared as appropriate) will be reported alongside *p*-values throughout.

**Screening and sensitivity analyses:** Total weekly physical activity will be truncated at 28 h/week and combined daily screen time at 16 h/day, consistent with prior ATLS protocols (20, 30, 32, 33). Sensitivity analyses will assess the impact of: (1) missing data, using multiple imputation where the proportion of missing values exceeds 5%; (2) data truncation rules; and (3) exclusion of extreme anthropometric values. Statistical significance will be set at *p* ≤ 0.05 for all confirmatory analyses.

## 3 Discussion

This protocol presents a rigorous and comprehensive approach to examining lifestyle behaviors, dietary patterns, sleep, sedentary time, and health outcomes among adolescents in Kuwait. It builds directly on the Kuwait component of the Arab Teens Lifestyle Study (ATLS), making this the first study to revisit adolescent obesity and its relationship with lifestyle behaviors using the same methodological framework applied in 2009/2010. Maintaining the original sampling strategy ensures high comparability across time, while the addition of accelerometer-based measures, grip strength, and psychological wellbeing substantially expands the scope of inquiry.

A major contribution of this study is its ability to fill the critical surveillance gap left by the KNSS. Although the KNSS remains the country's primary nutrition surveillance platform, it provides limited detail on adolescents, particularly regarding lifestyle determinants of obesity, dietary habits, sleep, sedentary behavior, and mental wellbeing. By contrast, the present study offers a detailed behavioral, psychological, and physiological profile of adolescents dimensions that are almost entirely absent from the KNSS. This protocol therefore fills an essential evidence gap and aligns with Kuwait's need for age-specific, actionable data to guide youth-focused public health strategies.

Several methodological strengths enhance the robustness of the study. The use of a large, stratified, and nationally representative sample across all six governorates ensures generalisability to Kuwaiti adolescents. The combination of subjective (questionnaires) and objective (accelerometery) assessments provides a more accurate and reliable picture of daily movement, sedentary time, and sleep than self-report alone. The use of validated tools, including the ATLS questionnaire, ASBQ, and BPWBS, alongside anthropometry and grip strength, offers a multidimensional assessment of adolescent health. In addition, careful scheduling to avoid Ramadan and other periods known to alter sleep and activity patterns reduces seasonal and cultural bias. The application of post-stratification survey weights and cluster-adjusted analyses further strengthens the representativeness and inferential validity of the findings.

The findings generated from this study will provide the first comprehensive update on adolescent lifestyle behaviors and obesity in Kuwait since the 2009/2010 ATLS. By enabling a direct comparison across a 15-year period marked by profound socioeconomic and technological change, the study will shed light on secular changes in physical activity, screen time, diet, sleep, and mental wellbeing. Importantly, the dataset will complement rather than duplicate the KNSS, filling gaps in behavioral, psychological, and objective activity data and offering a nuanced understanding of the factors driving adolescent obesity.

These insights will support evidence-based school health programmes, inform Ministry of Health and Ministry of Education policies targeting youth physical activity and nutrition, and contribute to a stronger national surveillance framework. At a regional level, the study will enhance the Gulf's limited adolescent health evidence base and may serve as a model for establishing long-term monitoring systems tailored to the unique cultural and environmental context of Kuwait.

### 3.1 Strengths

First, the study replicates the design and sampling strategy of the original Kuwait component of the ATLS (2009/2010) (9, 29), enabling direct comparison over a 15-year period. Such temporal comparability is rare in Kuwait and will allow a clear examination of secular changes in adolescent obesity and lifestyle behaviors. Second, the inclusion of a large, stratified, and representative sample drawn from public and private secondary schools across all six governorates enhances the generalisability of the findings. Third, the study adopts a multidimensional assessment strategy, combining self-reported data (ATLS, ASBQ), objective measures (accelerometers), anthropometric indices, grip strength, and validated psychological wellbeing tools. Fourth, the use of survey-adjusted analyses incorporating post-stratification weights addresses the complex sampling design and ensures nationally representative inferences.

### 3.2 Limitations

Despite these strengths, some limitations should be acknowledged. As in all school-based surveys, reliance on self-reported questionnaires may introduce recall or social desirability bias for dietary habits, physical activity, sedentary time, and psychological wellbeing. Although accelerometers provide objective indicators of movement behaviors, their use is dependent on funding and participant compliance. Additionally, the cross-sectional design precludes causal inference; observed associations cannot be interpreted as directional cause–effect relationships. However, cross-sectional evidence remains essential for informing surveillance, hypothesis generation, and future prospective studies. The protocol does not include a direct assessment of cardiorespiratory fitness, such as the 20-meter shuttle run, which would have provided an important additional indicator of adolescent physical health and cardiometabolic risk. This omission reflects the logistical constraints of conducting standardized fitness testing across 42 school classes spanning six governorates; future surveillance studies building on this protocol are encouraged to incorporate this measure. The maturity offset method, while objective and culturally appropriate, was developed primarily in White European populations and may carry some prediction error when applied to Arab adolescents. Finally, excluding data collection during Ramadan and other periods of known behavioral confounding is necessary to reduce bias, but may slightly limit seasonal generalisability.

The project will contribute directly to strengthening national capacity in adolescent health research and surveillance within the Ministry of Health. Workshops will be delivered by national investigators and international collaborators with expertise in adolescent epidemiology, movement-behavior assessment, and nutrition surveillance. These activities will promote long-term knowledge transfer and help establish a sustainable foundation for future public health research initiatives in Kuwait.

### 3.3 Dissemination

The findings of this study will be disseminated through multiple channels to ensure maximum scientific, policy, and community impact. Results will be shared in final project reports and presented at national and international conferences focusing on adolescent health, physical activity, nutrition, and public health. Manuscripts will be prepared for submission to peer-reviewed scientific journals, ensuring broad academic reach and long-term accessibility.

This research is both interdisciplinary and cross-disciplinary, integrating expertise from nutrition, physical activity, public health, psychology, behavioral epidemiology, and built-environment research. Its comprehensive design will support a wide range of dissemination outputs tailored to different stakeholders, including policymakers, school administrators, teachers, and health practitioners.

The project is also expected to strengthen research capacity in Kuwait. Team members, including junior researchers, dietitians, and assistants, will gain hands-on experience in accelerometer-based data processing, survey administration using digital platforms, field measurement protocols, and statistical analysis. In addition, the study will generate new evidence on built-environment features and their associations with adolescents' movement behaviors, dietary patterns, and health outcomes, informing the development of context-specific interventions and policies.
